# High-throughput-compatible assays using a genetically-encoded calcium indicator

**DOI:** 10.1038/s41598-019-49070-8

**Published:** 2019-09-03

**Authors:** Nyantsz Wu, Walter K. Nishioka, Noël C. Derecki, Michael P. Maher

**Affiliations:** Janssen Research & Development, LLC, San Diego, CA 92121 USA

**Keywords:** Fluorescent proteins, High-throughput screening, Receptor pharmacology

## Abstract

Measurement of intracellular calcium in live cells is a key component of a wide range of basic life science research, and crucial for many high-throughput assays used in modern drug discovery. Synthetic calcium indicators have become the industry standard, due their ease of use, high reliability, wide dynamic range, and availability of a large variety of spectral and chemical properties. Genetically-encoded calcium indicators (GECIs) have been optimized to the point where their performance rivals that of synthetic calcium indicators in many applications. Stable expression of a GECI has distinct advantages over synthetic calcium indicators in terms of reagent cost and simplification of the assay process. We generated a clonal cell line constitutively expressing GCaMP6s; high expression of the GECI was driven by coupling to a blasticidin resistance gene with a self-cleaving *cis*-acting hydrolase element (CHYSEL) 2A peptide. Here, we compared the performance of the GECI GCaMP6s to the synthetic calcium indicator fluo-4 in a variety of assay formats. We demonstrate that the pharmacology of ion channel and GPCR ligands as determined using the two indicators is highly similar, and that GCaMP6s is viable as a direct replacement for a synthetic calcium indicator.

## Introduction

Calcium ions (Ca^2+^) serve as a ubiquitous second messenger within cells across all domains of life^1^, modulating a wide array of cellular processes. Hundreds of proteins have evolved to modulate intracellular calcium levels, and to transduce changes in calcium concentrations into downstream cellular signals. Monitoring intracellular calcium levels is a valuable technique for modern drug discovery, allowing functional measurements of target proteins and calcium-mediated physiological pathways. The first molecular probe used to measure intracellular calcium was the genetically-encoded calcium indicator (GECI) aequorin, a bioluminescent protein derived from the jellyfish *Aequorea victoria*^2^. While aequorin has been successfully adapted for use in high-throughput screening (HTS), widespread adoption has been limited by the requirement for the use of exogenous coelenterazine and the complex calcium- and time-dependence of the response. Since the discovery of the first synthetic calcium indicators by Tsien^3^, based upon the coupling of a fluorophore to a calcium chelator, multiple efforts to fine-tune affinity, selectivity, and optical properties have generated a large panel of probes (reviewed by Paredes *et al*.^4^). Their brightness, photostability, and large dynamic range have allowed synthetic calcium indicators to become the probes of choice for high-throughput calcium assays.

Using molecular biology tools in place of synthetic chemistry, fluorescent GECIs were created by attaching fluorophores (variants of green fluorescent protein (GFP)) to a calcium binding motif (calmodulin)^5,6^. The most significant advantage of GECIs relative to synthetic calcium indicators is that they do not require dye loading; the cells themselves create the indicator. Over the past two decades, *in vivo* applications in neuroscience research have been the primary drivers for optimization of calcium affinity, response speed, and fluorescence properties of GECIs^7,8^. Recent reviews provide historical overviews of the discovery and optimization of GECIs^7,9,10^. The current set of GECIs are now comparable in these properties to synthetic indicators. A wide variety of single-wavelength GECIs have been characterized, including the GCaMPs^11^, pericams^12^, GECOs^13^, camgaroos^14^, and CatchERs^15^. Several classes of ratiometric indicators have also been discovered, including the emission-shift probe GEM-GECO1^13^ and variants, and dual-fluorophore FRET probes such as the cameleons^6,16^, the TN series^17,18^, and the Twitch series^19^. Compared to single-wavelength probes, ratiometric indicators generally offer improved dynamic range, the ability to correct for variations in volume or expression differences in individual cells, and the ability to determine absolute rather than relative calcium concentration^20^.

In addition to their utility *in vivo*, the improved GECIs are well suited as replacements for synthetic calcium indicators for *in vitro* applications. With respect to high-throughput assays (defined here as parallelized 96-, 384-, or 1536-well formats with the potential for automation to >10,000 individual data points per day), the use of a GECI in place of a synthetic calcium indicator would remove one or more time-consuming and expensive step(s). GECIs provide additional advantages over synthetic probes, including the ability to tag specific proteins, label individual subcellular compartments, and the possibility of using standard molecular biology techniques to modify the properties of the probes (see Discussion for more details).

Despite clear advantages of fluorescent GECIs, few reports have been published on their use in assays suitable for high-throughput screening. Cai *et al*.^21^ demonstrated HTS-compatible assays for ion channels and GPCRs using transient transfection of a modified GCaMP3 into HEK293 cells. In this study, cysteine pairs were added to the fluorescent protein to promote oxidation in metabolically-compromised cells, improving dynamic range by reducing background fluorescence from dead or dying cells. Honarnejad *et al*.^22^ described a high-content calcium screen designed to detect dysregulation of endoplasmic reticulum (ER) using stably-expressed YC3.6 GECI in HEK293. Murayama *et al*. used a GECI targeted to ER^23^ to screen for ryanodine receptor inhibitors^24^.

Several barriers have impeded the widespread adoption of GECIs by the screening community, including the following. (1) Direct comparison in cultured cells showed inferior sensitivity and effective dynamic range of the GECIs compared to synthetic probes (e.g. Lock *et al*.^25^). (2) Stable cell lines generally show low expression levels. (3) Most GFP variants have complex intellectual property issues. (4) Cytotoxicity, nuclear accumulation of the probes, and altered cellular responsivities have been observed in *in vivo* applications (reviewed by Rose *et al*.^26^). Here, we describe a platform based upon stable expression of a licensable GECI that provides a plug-and-play solution as a direct replacement for synthetic calcium dyes for HTS applications.

The GCaMP family of GECIs comprises some of the brightest, most-stable single-wavelength GECIs currently available. These probes are based upon circularly-permuted GFP variants coupled to modified versions of calmodulin and the M13 peptide from myosin light chain kinase^11^. The binding of calcium allows calmodulin/M13 to fold tightly against GFP, greatly increasing fluorescence by closing a water-accessible gap in the side of the GFP module^27^. Multiple rounds of optimization for speed, sensitivity, and dynamic range led to the GCaMP6 probes^28^. Here, we work with GCaMP6s, the member of the family with the highest calcium affinity^28^.

To maximize the utility of the platform, we preferred stable expression of the GECI. Our initial attempts to use GECI constructs for high-throughput compatible assays using transient transfections of unmodified GCaMP6s resulted in adequate transfection efficiency and fluorescence intensity. We were concerned that the potential cytotoxicity associated with long-term expression of GECIs would provide a negative selection pressure on a clonal cell line made using standard plasmids, in which the antibiotic selection marker is physically separated from the gene of interest (GOI). The progeny of a cell which disables the expression of the GOI while retaining the selection marker could gain a competitive advantage, gradually taking over the culture and limiting the useful lifetime of the clonal population. Therefore, we coupled the DNA encoding GCaMP6s to the DNA encoding a Blasticidin-S resistance (Bsr) gene^29^, using the self-cleaving porcine teschovirus 2A sequence^30^. The 2A linker allows for near-stoichiometric expression of two separate proteins driven by a single promoter. The progeny of a cell that disables the GOI in this system will also lose the expression of the selection marker, and quickly die off. Bsr is a member of the cytidine deaminase family and functions as a tetramer. Production of tetramers for efficient selection against Blasticidin S forces increased expression of the overall fusion protein^29,31^, and should improve suitability for HTS-compatible assays. Here, we describe the validation of HTS-compatible assays using stable expression of GCaMP6s for ion channel and GPCR targets.

## Results

### Transient transfection of GCaMP6s

**Figure 1:**
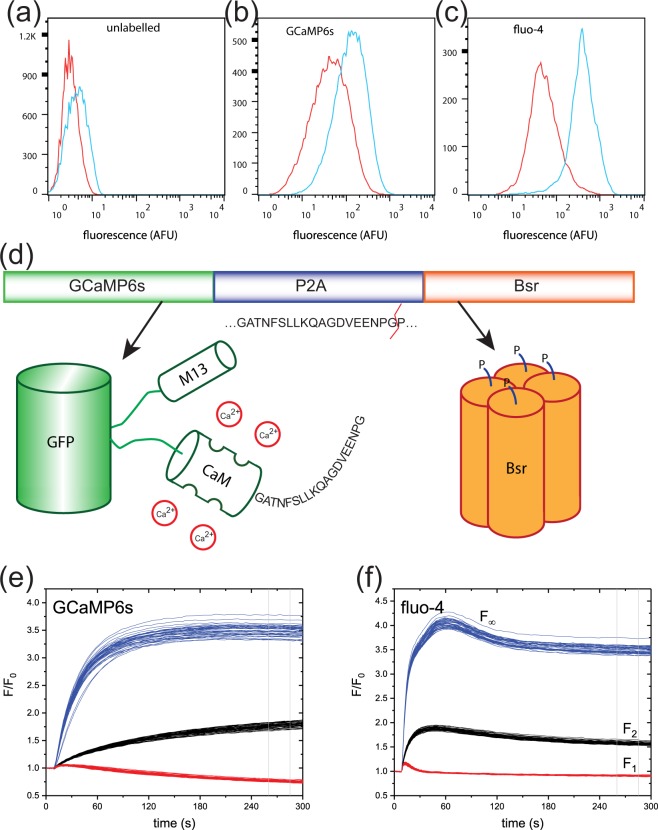
Preliminary comparisons of 293-F cells labelled with GCaMP6s or with fluo-4. (**a**–**c**) Histogram of log-transformed fluorescence intensity by flow cytometry for (**a**) unlabelled cells, (**b**) transient expression of pGP-CMV-GCaMP6s, and (**c**) cells labelled with fluo-4. Cells were treated with buffer (red) or with 10 µM ionomycin (blue). (**d**) Schematic representation of the GCaMP6s-P2A-Bsr construct. During translation, the P2A linker fails to form a peptide bond between glycine and proline. GCaMP6s and Bsr emerge as separate proteins, with fragments of the P2A linker at the C- and N-termini as shown. (**e**,**f**) Time-course of fluorescent signal for stable 293F.GluA1o-γ4 during stimulation with 15 µM glutamate. (**e**) Transiently-transfected GCaMP6s-2A-blast. (**f**) Labelled with fluo-4. Black lines indicate n = 40 negative control wells (0.5% DMSO only); blue lines indicate n = 40 control wells pre-incubated with a PAM (10 µM LY-395153); red lines indicate n = 40 positive control wells pre-incubated with an inhibitor (50 µM CP-465022). Vertical grey lines indicate the position of the measurement window.

GCaMP6s and fluo-4 have similar excitation and emission spectra (see Table 1), allowing direct comparison using fluorescein filter sets. For our initial characterization of GCaMP6s, we used flow cytometry to compare the fluorescence intensity of 293-F cells labelled with fluo-4 to cells 24 hours after transfecting with the pGP-CMV-GCaMP6s^28^ construct (Figure 1a–c). Identical settings for the flow cytometer were used for all conditions, allowing for direct comparison of the intensities. Intensities were measured in Arbitrary Fluorescence Units (AFU). The histograms of the background fluorescence of unlabelled cells are shown in Fig. 1a. For cells labelled with fluo-4, the log-scale histogram of the fluorescence intensity of cells tested in buffer was well-fitted by a single gaussian distribution with a center at 6.2 AFU. Fluo-4-labelled cells treated with ionomycin, a calcium ionophore which allows free entry of calcium, showed a single peak at 410.0 AFU. 293-F cells transiently transfected with pGP-CMV-GCaMP6s and tested in buffer showed a peak centered at 44.5 AFU. When treated with ionomycin, the histogram of the GCaMP6s-transfected cell population showed a peak at 133.3 AFU.

**Table 1:** Comparison of biophysical properties of calcium indicators used in this work. Values were derived from measurements by Chen *et al*.^28^ for GCaMP6s and by Gee *et al*.^57^ for fluo-4, in *in vitro* (cell-free) environments under saturating calcium conditions at neutral pH. Parameters are λ_max_ (ex): wavelength of fluorescence excitation maximum; λ_max_ (em): wavelength of emission maximum; ε_max_: absorption coefficient at the excitation maximum; QY: fluorescence quantum yield; EC_50_: midpoint of calcium binding curve; Hill slope for the calcium binding curve; k_off_: off-rate for calcium unbinding; DR: fluorescence dynamic range from zero to saturating calcium concentration.

	GCaMP6s^28^	Fluo-4^57^
λ_max_ (ex) (nm)	497	494
λ_max_ (em) (nm)	515	516
ε_max_ (M^−1^cm^−1^)	68500	88000
QY	0.61	0.14
EC_50_ (nM)	144	345
Hill slope	2.9	1.0
k_off_ (s^−1^)	1.1	369^a^
DR (F_∞_/F_0_)	63	>100

^a^measured for fluo-3^58^.

With the goal of ensuring long-term expression stability of GCaMP6s in a clonal cell line, we generated an expression plasmid in which the drug selection gene is directly coupled to the GCaMP6s sequence, using a spontaneously-cleaving 2A linker. Figure 1d shows a schematic representation of the GCaMP6s-P2A-Bsr construct. GCaMP6s is a circularly-permuted GFP variant with an M13 domain on the N-terminus, and a modified calmodulin (CaM) domain on the C-terminus. As the protein is being expressed, residues from the P2A linker are added to the C-terminus of GCaMP6s. At the end of the P2A sequence, interactions between the emerging protein interact with the ribosome, preventing the formation of the terminal gly-pro peptide bond^32^. Translation continues into the Bsr sequence despite the failure of this bond to form. The end result is the expression of two completely separate proteins, with a 19-amino acid fragment appended to the C-terminus of the GCaMP6s and an extra proline at the N-terminus of the Bsr protein. Bsr functions as a tetramer; this system drives the expression of four GCaMP6s subunits for each functional Bsr complex.

We tested this construct using a calcium flux assay based on a clonal cell line stably expressing a fusion of human GluA1o with human TARP-γ4 in 293-F cells. The α-amino-3-hydroxyl-5-methyl-4-isoxazole-propionic acid (AMPA) subtype of ionotropic glutamate receptors are ligand-gated ion channels that mediate the majority of fast synaptic transmission within the mammalian brain. AMPA receptors (AMPARs) comprise tetramers of pore-forming GluA subunits in complex with a variety of accessory proteins. Although AMPARs lacking RNA-edited GluA2 subunits are calcium-permeant, homotetramers of GluA1o desensitize almost completely after a few milliseconds of exposure to agonist. Since FLIPR assays typically operate on the timescale of 1–100 seconds, cells expressing GluA1o alone do not produce detectable responses. Co-expression of members of the transmembrane AMPAR regulatory protein (TARP) family, including TARP-γ4, reduces desensitization sufficiently to allow measurable calcium flux. Figure 1f shows fluorescence as a function of time as measured in FLIPR for control wells, using the synthetic calcium indicator fluo-4. Wells stimulated with 15 µM glutamate alone showed a steady-state response of F_2_/F_0_ = 1.53 ± 0.05 (mean ± SD, n = 128 wells). To estimate the full effective dynamic range of the indicator, we measured the fluorescence in near-saturating calcium concentrations using an addition of 15 µM glutamate plus 10 µM LY-395153, a positive allosteric modulator (PAM) which prevents desensitization of the AMPA receptors. Under these conditions the fluorescence rose to a maximum of F_∞_/F_0_ = 4.04 ± 0.10, followed by a decline to a steady-state response of F/F_0_ = 3.47 ± 0.08 (mean ± SD, n = 128 wells). The fluorescence under full inhibition of the AMPA receptor was measured using a simultaneous addition of 15 µM glutamate plus 50 µM CP-465022. After a small, transient rise, the fluorescence dropped to a steady-state response of F_1_/F_0_ = 0.89 ± 0.01 (mean ± SD, n = 128 wells).

We performed a transient transfection of GCaMP6s-P2A-Bsr in the GluA1o-γ4 cell line. Figure 1e shows the time course of fluorescence for control wells using these cells, using the same format as for GluA1o-γ4 cells labelled with fluo-4. Wells stimulated with 15 µM glutamate alone had a steady-state response of F_2_/F_0_ = 1.87 ± 0.05 (mean ± SD, n = 128 wells). The working dynamic range of the indicator, using an addition of 15 µM glutamate plus 10 µM LY-395153, was F_∞_/F_0_ = 3.41 ± 0.34 (mean ± SD, n = 128 wells). The fluorescence under full inhibition of the AMPA receptor was F_1_/F_0_ = 0.70 ± 0.07 (mean ± SD, n = 128 wells).

### Stable expression of GCaMP6s

We placed under blasticidin selection an aliquot of the GluA1o-γ4 cells that had been transfected with GCaMP6s-P2A-Bsr, and isolated single cells to select for a clonal cell line stably expressing both GluA1o-γ4 and GCaMP6s (designated 293F.GluA1o-γ4.GCaMP6s.blast). We also generated a clonal cell line in wild-type 293-F by transfecting with GCaMP6s-P2A-Bsr, followed by selection with blasticidin and isolation of single cells; this cell line is designated 293F.GCaMP6s.blast.

#### AMPA receptors

**Figure 2:**
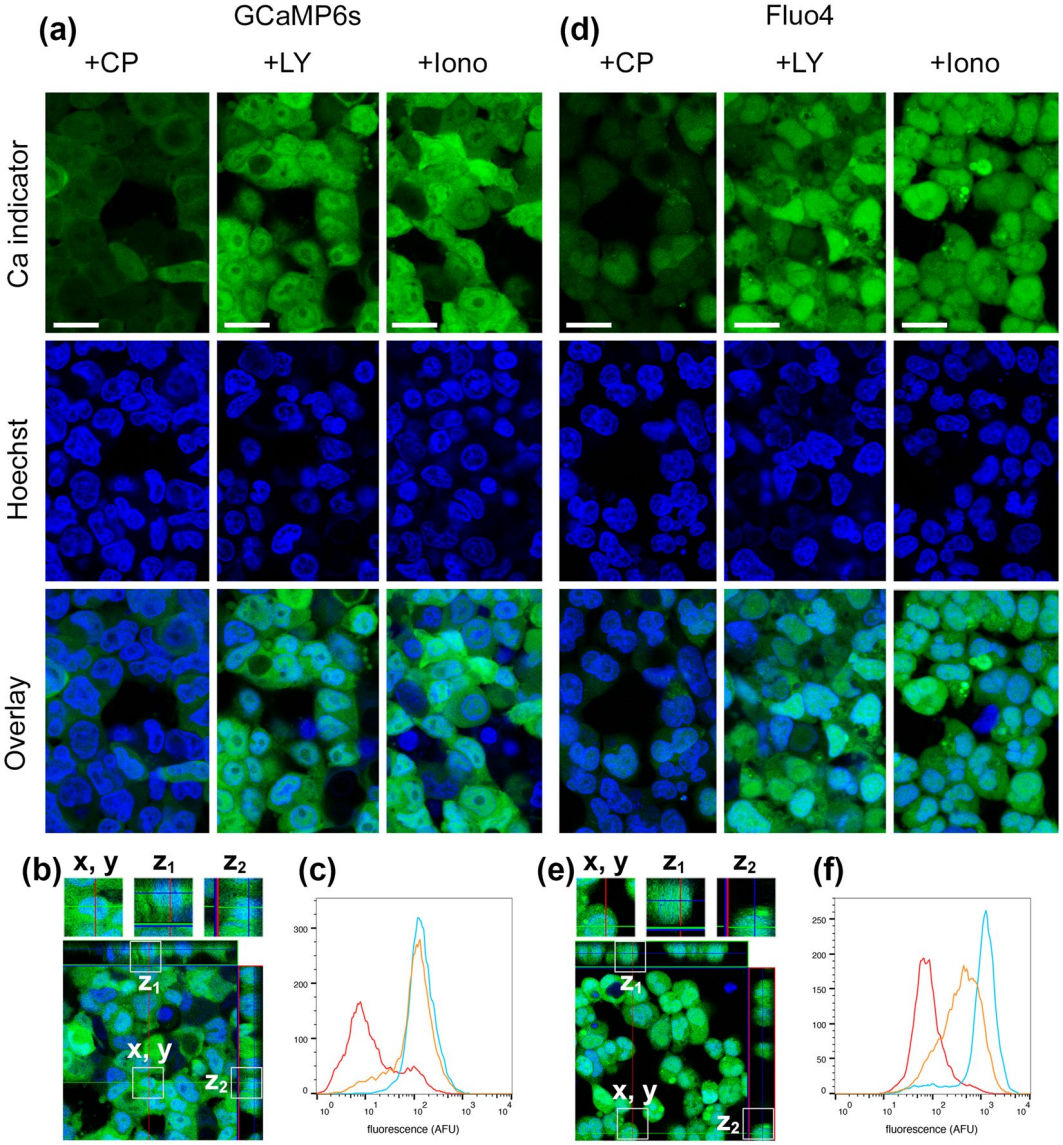
Images and cytometry of stably-expressed GluA1o-γ4 293-F cells. Cells were labelled with (**a**–**c**) stable expression of GCaMP6s and (**d**–**f**) fluo-4. Pseudocolors represent the intensity with 488 nm excitation (green; GCaMP6s or fluo-4) and 405 nm excitation (blue; Hoechst 33342). (**a**,**d**) First column in each group: cells treated with 10 µM CP-465022 to fully inhibit AMPAR-mediated calcium flux. Second column in each group: cells treated with 10 µM glutamate and 10 µM LY-395153. Third column in each group: cells treated with 10 µM ionomycin to fully saturate the indicator in all labelled cells. The first row shows the fluorescence intensity at 488 nm excitation. The second row shows the fluorescence intensity at 405 nm excitation. The third row is an overlay of the first and second rows. Scale bars are 20 µm. (**b**,**e**) Vertical projections of overlay images. The narrow side panels at the top and right edges of the main image show vertical projections along the faint overlay lines. The small square image at the top is a magnification of the box marked ‘x, y’ in the main image. The adjacent square images show the z-axis projections of this box. (**c**,**f**) Histograms of fluorescence intensity by flow cytometry of cells in suspension: CP-465022 (red), glutamate plus LY-395153 (orange), ionomycin (blue).

We compared the cellular and subcellular distributions of stably-expressed GCaMP6s in 293F.GluA1o-γ4.GCaMP6s.blast cells to the parental 293F.GluA1o-γ4 cells labelled with fluo-4, using confocal microscopy. Figure 2a shows representative images of 293F.GluA1o-γ4.GCaMP6s.blast cells. These cells were also labelled with the nuclear stain Hoechst 33342 (blue). Laser power and detector settings were identical for all of the images in Fig. 2a, so these images are directly comparable. In the presence of 10 μM CP-465022 (used to fully inhibit calcium flux due to residual glutamate), GCaMP6s fluorescence was uniformly dim. Dead cells, identified by morphology, were relatively sparse and showed no GCaMP6s labelling. In the presence of 15 µM glutamate plus 10 µM LY-395153 or 10 μM ionomycin, most cells show bright, uniform cytoplasmic fluorescence with minimal punctate staining. Cell nuclei appear dimmer than the cytoplasm, suggesting nuclear exclusion of GCaMP6s. This phenomenon is more clearly seen in the expanded views and vertical projections of individual cells in Fig. 2b. There is very little overlap of green and blue staining in the vertical projections. The cells used for these images had been in continuous culture for six months and had therefore undergone ~180 cell divisions.

Figure 2d shows representative images of 293F.GluA1o-γ4 cells labelled with fluo-4. Laser power and detector settings were reduced relative to the cells labelled with GCaMP6s, as the fluorescence intensity for the fluo-4 labelled cells was substantially brighter (see cytometry results for quantification). Similar to the cells expressing GCaMP6s, cells labelled with fluo-4 showed very dim green fluorescence in the presence of CP-465022 and bright cytoplasmic fluorescence in the presence of glutamate/LY-395153 or ionomycin. Unlike GCaMP6s, fluo-4 is present in the cell nuclei and shows increased intensity in other organelles. While some vacuoles in the fluo-4-labelled cells appear to lack green fluorescence, the nuclei appear to be co-labelled with fluo-4 and Hoechst 33342. In the expanded views and vertical projections in Fig. 2e, green fluorescence clearly overlaps with the nuclear staining.

Histograms of the logarithmic fluorescence intensity of individual cells as determined by flow cytometry are shown in Fig. 2c for GCaMP6s and Fig. 2f for fluo-4. Flow cytometer settings were the same as for the measurements shown in Figure 1a–c, allowing for direct comparison of the intensities. GCaMP6s cells treated with ionomycin show a single peak at 139 AFU, indicating that essentially the entire population is uniformly labelled. For treated with CP-465022, two peaks are visible; based on gaussian fits, these have centers at 7.9 AFU (75% of the population) and 77.2 AFU (25% of the population). Two peaks are also visible for cells treated with glutamate/LY-395103, with centers at 18.4 AFU (18% of the population) and 127.6 AFU (82% of the population). Because all cells were uniformly labelled, the pharmacological treatments indicate that when all cells are averaged in a FLIPR-type experiment, 18% of the cells lack sufficient receptor to contribute to a response; an additional 25% of the cells will contribute to background fluorescence with reduced dynamic range available. Thus, only 57% of the cells from this clone can contribute fully to an agonist response.

Fluo-4-labelled cells treated with ionomycin showed a single peak at 1247 AFU containing 90% of the population; 10% of the cells had fluorescence intensities less than 300 AFU in a broad featureless distribution. Fluo-4-labelled cells treated with CP-465022 show two peaks, with centers at 71 AFU (87% of the population) and 257 AFU (13% of the population). Two peaks are also visible for cells treated with glutamate/LY-395103, with centers at 119 AFU (25% of the population) and 552 AFU (75% of the population). Similar to the situation for the cells labelled with GCaMP6s, only approximately 62% of the cells from this clone can contribute fully to an agonist response.

**Figure 3:**
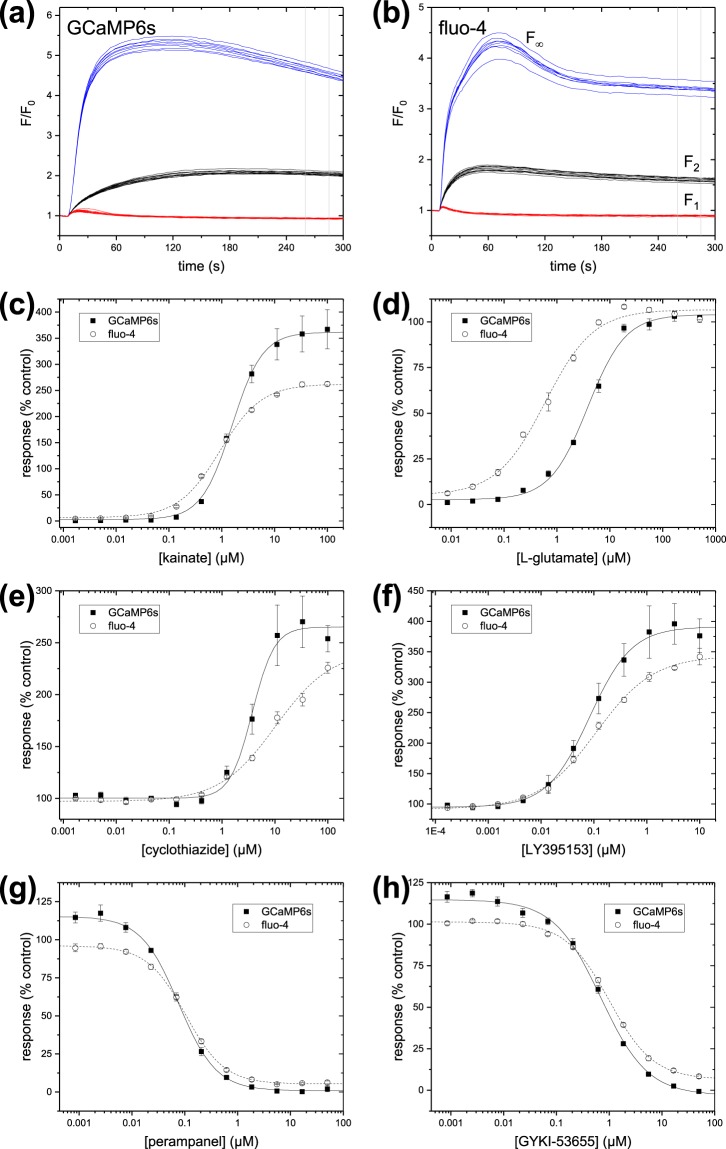
Comparison of intracellular calcium assays for stably-expressed GluA1o-γ4. Time-courses during stimulation with 15 µM glutamate for cells labelled with (**a**) stable expression of GCaMP6s and (**b**) fluo-4. Black lines indicate n = 12 negative control wells (0.5% DMSO only); blue lines indicate n = 8 control wells pre-incubated with a PAM (10 µM LY-395153); red lines indicate n = 12 positive control wells pre-incubated with an inhibitor (50 µM CP-465022). Vertical grey lines indicate the position of the measurement window. (**c**–**h**). Normalized response as a function of concentration of test compounds as indicated in the legends. Data are expressed as mean and standard deviation of all individual data points from three separate experiments. Lines are Hill function fits to the data.

The cytometry experiments allow direct comparison of the fluorescence intensities between the two indicators. FLIPR-type experiments measure the total fluorescence intensity of 10,000–20,000 cells, which can be emulated by the arithmetic mean in a cytometry run of ~10,000 cells. A summary of multiple cytometry measurements is shown in Table 2. The ionomycin treatment indicates that the total fluorescence output of GCaMP6s-labelled cells was 6.8X lower than cells labelled with fluo-4; the fluorescence in resting calcium conditions (cells treated with CP-465022) was 3.0X lower in GCaMP6s-labelled cells than in cells labelled with fluo-4. Although the fluorescence output of GCaMP6s-labelled cells is substantially dimmer compared to fluo-4, they are sufficient to give robust responses in the FLIPR experiments (compare Fig. 3a,b).

**Table 2:** Summary of cytometry experiments. For each experimental run, the arithmetic mean of all cells larger than 9 µm diameter was measured (1,800–11,000 cells per run). Data are expressed as mean ± standard deviation of the results from each run. The number of experimental runs is shown in parentheses. Cell treatments were CP-465022 (F_CP_); glutamate plus LY-395153 (F_PAM_); ionomycin (F_iono_). ΔF is (F_PAM_ -F_CP_) or (F_iono_ -F_CP_).

	GCaMP6s		Fluo-4	
	mean	DR_eff_	mean	DR_eff_
	(AFU)	ΔF/F_CP_	(AFU)	ΔF/F_CP_
F_CP_	42 ± 8 (4)		127 ± 17 (5)	
F_PAM_	132 ± 5 (4)	2.14	424 ± 76 (4)	2.33
F_iono_	177 ± 2 (4)	3.2	1211 ± 31 (4)	8.51

We measured the apparent potency of a panel of AMPA receptor agonists, antagonists, and PAMs to compare the performance of stably-expressed GCaMP6s in 293F.GluA1o-γ4.GCaMP6s.blast cells, to the parental 293F.GluA1o-γ4 cells labelled with fluo-4. Using single-addition assays, we monitored the fluorescence in a 384-well plate format as cells were exposed to a range of concentrations of test compounds generated by serial dilution. The assay buffer contained 4 mM calcium. For antagonists and PAMs, test compounds were pre-incubated for 60 minutes prior to the assay, and the cells were challenged with 15 µM glutamate. All compounds were tested in duplicate. Time-courses for the control wells are shown in Fig. 3a for cells stably expressing GCaMP6s, and Fig. 3b for cells labelled with fluo-4. The time-course and magnitude of fluorescence response to agonist were similar for the two calcium indicators. The responses in wells treated with positive antagonist controls (red), positive PAM controls (blue), and negative control wells (black) showed modest scatter and excellent separation for both indicators. The PAM positive control wells were treated with 10 μM LY-395153, which prevents desensitization of AMPA receptors; the intracellular calcium in these wells is expected to approach saturation for the calcium indicators.

The normalized responses as functions of concentration for representative agonists, antagonists, and PAMs are shown in Fig. 3c–h. Three separate concentration-response experiments were performed using cells labelled with fluo-4, or with stably-expressed GCaMP6s. The mean potency of n = 3 measurements of the compounds in each assay format are shown in Table 3. Statistics describing the assay performance for individual plates are summarized in Table 4.

**Table 3:** Comparison of pharmacology for test compounds in the GluA1o-γ4 intracellular calcium assays. Data are expressed as the mean ± standard deviation of n = 3 individual concentration-response experiments.

Name	Type	GCaMP6s pEC_50_	Fluo-4 pEC_50_
kainate	agonist	5.84 ± 0.16	6.10 ± 0.09
glutamate	agonist	5.41 ± 0.03	6.27 ± 0.03
domoate	agonist	7.00 ± 0.12	7.34 ± 0.11
(S)-AMPA	agonist	6.26 ± 0.03	6.99 ± 0.14
(R)-AMPA	agonist	5.11 ± 0.13	5.81 ± 0.12
cyclothiazide	PAM	5.31 ± 0.05	5.02 ± 0.25
LY395153	PAM	7.08 ± 0.04	6.99 ± 0.26
		**GCaMP6s pIC**_**50**_	**Fluo-4 pIC**_**50**_
CP-465022	antagonist	6.73 ± 0.33	6.77 ± 0.15
perampanel	antagonist	7.10 ± 0.14	7.04 ± 0.10
talampanel	antagonist	6.02 ± 0.27	5.65 ± 0.08
NBQX	antagonist	6.14 ± 0.25	5.18 ± 0.06
GYKI53655	antagonist	6.16 ± 0.10	6.00 ± 0.13

**Table 4:** Summary of assay performance for individual assay plates in antagonist mode.

Target	GCaMP6s			Fluo-4		
	z′	DR_assay_ (F_2_ − F_1_)/F_1_	DR_eff_ (F_∞_ − F_1_)/F_1_	z′	DR_assay_ (F_2_ − F_1_)/F_1_	DR_eff_ (F_∞_ − F_1_)/F_1_
GluA1o-γ4	0.72	0.7	4.0	0.82	0.81	3.9
	0.82	1.1	5.0	0.81	0.80	3.8
	0.89	1.2	4.7	0.79	0.85	4.0
mAChR	0.68	1.7	4.4	0.66	0.44	2.8
	0.74	1.9	4.3	0.61	0.42	2.7
	0.73	1.7	3.8	0.63	0.44	3.0
TRPV1	0.63	2.0	3.6	0.88	3.8	5.9
	0.86	3.1	5.5	0.67	1.6	7.9
	0.76	2.7	4.7	0.68	0.82	6.4

#### Muscarinic acetylcholine receptors

Muscarinic acetylcholine receptors (mAChRs) are G-protein coupled receptors (GPCRs) with widespread tissue distribution and diverse physiological functions. G_q/11_-coupled mAChRs are expressed endogenously in HEK293 cells^33,34^, and serve as a convenient test system for intracellular calcium indicators. We measured the potencies of a panel of mAChR ligands in the 293F.GCaMP6s.blast cell line stably expressing GCaMP6s, compared to wild-type 293-F cells labelled with the synthetic indicator fluo-4. The assays were performed in 384-well plates in a single-addition assay format in FLIPR Tetra^®^; assay buffer contained 2 mM calcium. Test compounds were prepared in a serial dilution format, and each compound was tested in duplicate. For agonists, fluorescence was monitored as the test compounds were added. For antagonists, test compounds were incubated with the cells for 30 minutes prior to the assay, and fluorescence was monitored as the cells were challenged with acetylcholine.

**Figure 4:**
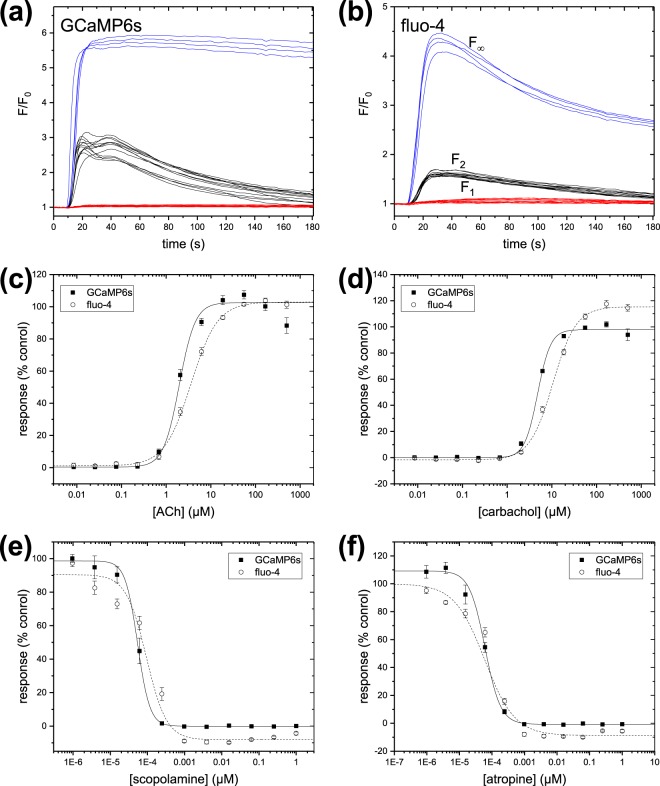
Comparison of intracellular calcium assays for the endogenous muscarinic receptor expressed in 293-F cells. Time-courses during stimulation with 30 nM ACh for cells labelled with (**a**) stable expression of GCaMP6s and (**b**) the synthetic indicator fluo-4. Black lines indicate n = 12 negative control wells (0.5% DMSO only); blue lines indicate n = 4 control wells treated with 10 µM ionomycin; red lines indicate n = 12 positive control wells pre-incubated with an inhibitor (1 µM scopolamine). (**c**,**d**) Normalized response as a function of concentration of selected antagonists. (**e**,**f**) Normalized response as a function of concentration of selected agonists. Data are expressed as mean and standard deviation of all individual data points from three separate experiments. Lines are Hill function fits to the data.

Time-courses for the control wells and normalized responses as functions of concentration for representative agonists and antagonists are shown in Fig. 4a,b. The time-course and magnitude of fluorescence response to agonist were similar for the two calcium indicators. The responses in wells treated with positive antagonist controls (red) and negative control wells (black) show modest scatter and excellent separation for both indicators. Because the muscarinic receptors are GPCRs and respond via calcium release from internal stores, the degree of saturation of the calcium indicator could not be determined directly through activation of the receptor. Therefore, we also included an additional set of control wells in which the calcium ionophore ionomycin was added during the first addition to fully saturate the calcium indicators (blue traces).

Three separate concentration-response experiments were performed using cells labelled with fluo-4, or with stably-expressed GCaMP6s. Normalized responses as functions of concentration for representative agonists and antagonists are shown in Fig. 4d–f. The mean potency of n = 3 measurements of the compounds in each assay format are shown in Table 5. Statistics describing the assay performance for individual plates are summarized in Table 4.

**Table 5:** Comparison of pharmacology for test compounds in the muscarinic receptor intracellular calcium assays. Data are expressed as the mean ± SEM of n = 3 individual concentration-response experiments.

Name	Type	Fluo-4 pEC_50_	GCaMP6s pEC_50_
acetylcholine	agonist	5.71 ± 0.08	5.47 ± 0.05
carbachol	agonist	5.33 ± 0.02	4.98 ± 0.02
oxotremorine	agonist	5.62 ± 0.02	5.31 ± 0.05
		**Ca5 pIC**_**50**_	**GCaMP6s pIC**_**50**_
atropine	antagonist	10.24 ± 0.13	9.99 ± 0.19
pirenzepine	antagonist	8.00 ± 0.16	7.59 ± 0.04
scopolamine	antagonist	10.26 ± 0.14	9.95 ± 0.24
4-DAMP	antagonist	10.26 ± 0.26	10.03 ± 0.15
methoctramine	antagonist	6.37 ± 0.23	6.28 ± 0.18

#### TRPV1

TRPV1 is a ligand-gated cation channel, whose activation by exogenous ligands such as capsaicin produce the burning sensation associated with hot peppers. TRPV1 is expressed in the central and peripheral nervous systems and serves a variety of physiological functions including pain perception and body temperature regulation. We used transient transfections of DNA encoding the guinea pig TRPV1 receptor in the 293F.GCaMP6s.blast cell line and in wild-type 293-F cells subsequently labelled with fluo-4, to compare the pharmacology of a panel of TRPV1 ligands. The assays were performed in 384-well plates in a single-addition assay format in FLIPR Tetra^®^. For antagonists, a range of concentrations of test compounds generated by serial dilution were pre-incubated on the cells for 60 minutes prior to the assay, and the cells were challenged with 10 µM capsaicin. For agonists, the test compounds were added on-line in place of the 10 µM capsaicin stimulus.

**Figure 5:**
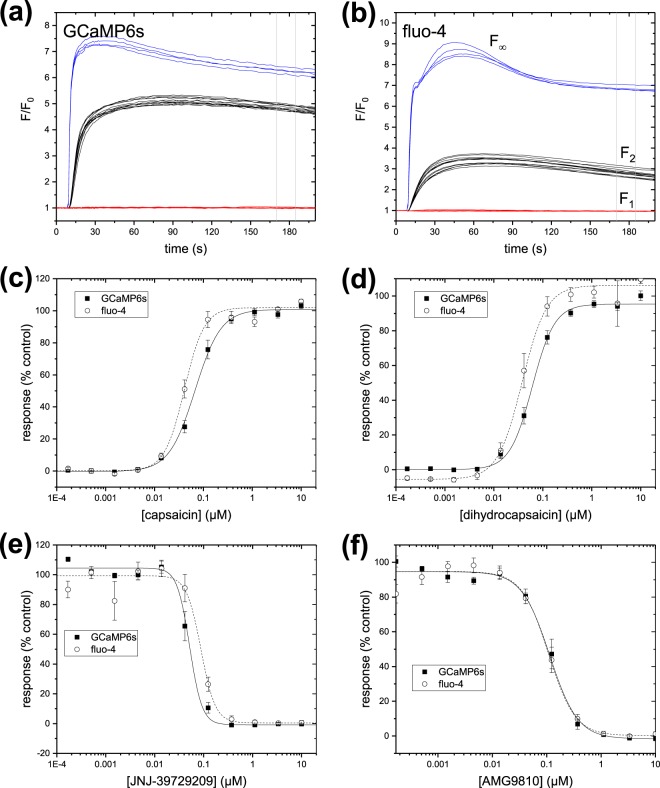
Comparison of intracellular calcium assays for TRPV1 receptors transiently expressed in 293-F cells. Time-courses for cells during stimulation with 150 nM capsaicin labelled with (**a**) stable expression of GCaMP6s and (**b**) the synthetic indicator fluo-4. Black lines indicate n = 12 negative control wells (0.5% DMSO only); blue lines indicate n = 4 control wells treated with 2 mM calcium; red lines indicate n = 12 positive control wells pre-incubated with an inhibitor (10 µM capsazepine). Vertical grey lines indicate the position of the measurement window. (**c**,**d**) Normalized response as a function of concentration of selected antagonists. (**e**,**f**) Normalized response as a function of concentration of selected agonists. Data are expressed as mean and standard deviation of all individual data points from three separate experiments. Lines are Hill function fits to the data.

Full activation of TRPV1 results in a large steady-state current, which can lead to saturation of a calcium indicator in physiological saline^35^ and subsequent shifts in apparent potency of test compounds^36^. These assays were performed in low calcium (20 µM) to avoid dye saturation. Time-courses for the control wells are shown in Fig. 5a,b. The time-course and magnitude of fluorescence response to agonist are similar for the two calcium indicators. The responses in wells treated with positive antagonist controls (red) and negative control wells (black) show modest scatter and excellent separation for both indicators. To estimate the degree of saturation of the calcium dye, we included control wells of 10 µM capsaicin with 2 mM calcium. Normalized responses as functions of concentration for representative agonists and antagonists are shown in Fig. 5a–f. The mean potency of n = 3 measurements of the compounds in each assay format are shown in Table 6. Statistics describing the assay performance for individual plates are summarized in Table 4.

**Table 6:** Comparison of pharmacology for test compounds in the TRPV1 intracellular calcium assays. Data are expressed as the mean ± SEM of n = 3 individual concentration-response experiments.

Name	Type	Fluo-4 pEC_50_	GCaMP6s pEC_50_
olvanil	agonist	7.68 ± 0.41	7.84 ± 0.35
arvanil	agonist	6.76 ± 0.51	7.12 ± 0.38
capsaicin	agonist	7.16 ± 0.07	7.40 ± 0.08
resiniferatoxin	agonist	6.91 ± 0.32	7.00 ± 0.59
*N*-oleoyl dopamine	agonist	5.63 ± 0.13	5.67 ± 0.26
dihydrocapsaicin	agonist	7.21 ± 0.12	7.44 ± 0.16
		**Fluo-4 pIC**_**50**_	**GCaMP6s pIC**_**50**_
JNJ-17203212	antagonist	6.71 ± 0.22	6.49 ± 0.39
BCTC	antagonist	7.87 ± 0.09	8.10 ± 0.29
SB-366791	antagonist	6.03 ± 0.21	5.84 ± 0.28
ruthenium red	antagonist	6.07 ± 0.13	6.92 ± 0.19
AMG9810	antagonist	6.82 ± 0.07	6.76 ± 0.07
JNJ-39729209	antagonist	7.18 ± 0.11	6.89 ± 0.14
capsazepine	antagonist	6.02 ± 0.12	5.65 ± 0.11

## Discussion

Based on flow cytometry, transient transfection of the genetically-encoded calcium indicator construct pGP-CMV-GCaMP6s into 293-F cells yielded a population of cells with a robust increase in fluorescence in the presence of ionomycin. This plasmid also contains a neomycin selection marker on a separate promoter. Upon selection of stably-expressing cells using G418, the expression levels of GCaMP6s were too low for consideration of selecting a clonal cell line bright enough for further use. Therefore, we coupled the DNA encoding GCaMP6s to a blasticidin resistance gene^29,37^ using a spontaneously-cleavable 2A linker^30^. This strategy forces the cell to fully express the target gene before being able to express the resistance marker^38^, enabling the generation of clonal cell lines with sufficient brightness and expression stability to support a high-throughput assay. Blasticidin deaminase is a tetramer of subunits, theoretically requiring the generation of four GCaMP6s proteins per functional enzyme. Further, we expect better long-term expression stability, since a cell cannot gain a competitive advantage by disabling expression of GCaMP6s.

After expression and spontaneous cleavage, fragments of the 2A linker remain on the C-terminus of the first gene (in this case, GCaMP6s) and the N-terminus of the second (the blasticidin resistance gene)^39^. Transient transfection of the GCaMP6s-P2A-Bsr into 293F cells expressing an AMPA receptor construct (GluA1o-γ4) yielded cells with strong, highly-stereotyped responses to glutamate addition and to ionomycin. This demonstrates that the 2A linker fragment on the GCaMP6s has a minimal effect on the function of the indicator. The noise level and effective dynamic range of cells labelled with GCaMP6s were similar to those labelled with fluo-4. For both indicators, the fluorescence of the antagonist positive control wells dropped slightly following the compound addition. In this case, agonist and antagonist were added at the same time. Residual glutamate in the bath is sufficient to partially activate the GluA1o-γ4 receptors prior to full agonist challenge, so that the intracellular calcium is already slightly elevated prior to agonist addition. This effect is more pronounced for GCaMP6s, since this indicator has a higher affinity for calcium compared to fluo-4.

While performing transient transfections is relatively simple and straightforward, one of the advantages of a genetically-encoded system is the ability to create stably-expressing clonal cell lines. Such a cell line would continuously manufacture its own indicator, requiring minimal intervention prior to the assay. Clonal cell lines based upon the GCaMP6s-P2A-Bsr construct showed bright, uniform cytoplasmic staining. Functional Blasticidin resistance indicates that the extra N-terminal proline on Bsr from the 2A linker had no major negative impact.

One of the problems experienced with the use of GECIs in *in vivo* applications is the tendency for accumulation of the indicator in the nucleus of neurons over a weeks-to-months timescale, leading to aberrant behavior of those cells^40^. Treatment of cells with the calcium ionophore ionomycin floods every compartment of the cell, showing the subcellular compartmentalization of the indicator. The 293F.GluA1o-γ4.GCaMP6s.blast cells used for the images in Fig. 2a,c had been in continuous culture for over six months, and showed no indication of nuclear accumulation. This is consistent with the cytoplasmic targeting of the indicator. A likely explanation for the difference between neurons and the 293-F cells is that any nuclear entry of GCaMP6s will be diluted amongst the progeny of the rapidly dividing 293-F cells, whereas terminally differentiated neurons do not undergo nuclear division. In contrast, all synthetic calcium indicators show labelling of intracellular compartments, including the nucleus, to varying degrees^41^.

High throughput-compatible assays, defined here as parallelized semi-automated experiments with the capacity for >10,000 data points per day, can be configured for multiple purposes that include screening libraries of compounds, determination of mechanism-of-action of test compounds, and measurement of functional potency at individual targets. Here, we focused on potency measurements, as this tends to be the most stringent assessment of the accuracy and reproducibility of an assay. We compared the performance of stably-expressed GCaMP6s to exogenously-applied fluo-4 in FLIPR Tetra^®^ assays, using cells expressing calcium-permeant ion channels or a G_q/11_-coupled receptor. The results were compared using multiple criteria: (1) reproducibility of the control responses, quantified by the assay quality parameter *z*′; (2) assay dynamic range, quantified by the ratio of the responses of the negative controls to the fully-inhibited controls *F*_2_*/F*_1_; (3) effective dynamic range of the dye, quantified by the calcium-saturated fluorescence divided by the fully-inhibited response *F*_*∞*_ /*F*_1_; and (4) comparison of potencies of standard compounds measured by fits to the response as a function of compound concentration.

For each receptor type, the assay quality parameter *z*′ > 0.5 for all plates tested for both indicators; this suggests high confidence in screening results. The effective dynamic range (maximal divided by minimal fluorescence intensity) ranged from 3.6 to 5.5 for GCaMP6s, similar to the range for fluo-4 (range of 2.7 to 8.0). The assay dynamic range (negative control fluorescence divided by the fluorescence for fully-inhibited controls) was also very similar between the two indicators.

The potencies of a panel of pharmacological agents targeting TRPV1 and the muscarinic receptor were comparable for the two indicators. Taken as a group, agonist potencies for AMPA receptors were 0.6 log units (3.7X) more potent, on average, using fluo-4 compared to GCaMP6s; antagonists were 0.3 log units (2X) less potent. Application of the Black-Leff operational model of agonism^42,43^, including the non-linear response of the calcium indicators as signal transduction elements, implies that the substantially different calcium sensitivities of the two indicators is involved (see Table 1). GCaMP6s has a calcium binding constant in buffer approximately 2.4X more potent than fluo-4, with a relatively steep Hill slope due to the tetravalent nature of the calmodulin. Although a full treatment is beyond the scope of this work, preliminary modeling suggests that the apparent potency measured by the two indicators under similar conditions can differ by 1- to 5-fold, and that agonists and antagonists will have shifts in opposite directions. Improved accuracy could potentially be achieved by careful calibration of the dyes, converting fluorescence measurements to intracellular calcium concentrations prior to the fitting process^36^.

The working dynamic range (measured as the maximal fluorescence achieved with near-saturating calcium divided by the level of fluorescence for a fully-inhibited receptor) was similar between cells labelled with GCaMP6s compared to those labelled with the synthetic calcium indicator, fluo-4 (Table 4). These dynamic ranges are substantially smaller than values reported under ideal conditions (see Table 1). Working dynamic range can be degraded from ideal by several factors, including background fluorescence of the cells and the multiwell plates, and the non-zero intracellular calcium levels at the beginning of the assay. With intracellular calcium levels measured at rest in HEK293 cells near 100 nM (see, for example, Patel *et al*.^44^), both indicators would be expected to have a non-zero basal fluorescence. While there are no established criteria for the minimal dynamic range required for a successful assay, these effective dynamic ranges were sufficiently large to enable acceptable assay quality for both indicators.

**Figure 6:**
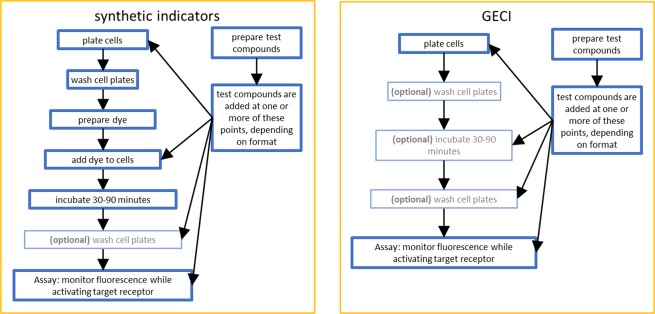
Representative workflows for calcium flux assays based on synthetic indicators or GECIs. Steps marked ‘optional’ may or may not be required, depending upon the specific requirements for the target of interest.

GECIs have numerous advantages relative to synthetic calcium indicators with respect to their use in high-throughput pharmacology assays, including the following. (1) Simplification of the assay procedure. Because the cells manufacture the indicator, exogenous application is unnecessary. Examples of typical workflow for the two types of indicators are shown in Fig. 6. In an HTS environment, every additional step decreases throughput and adds noise to the final result. (2) Reduced reagent cost. Having made the initial investment in creating a stable GECI cell line, no additional costs are involved with labelling cells. (3) While synthetic indicators must be created and purified on a large scale by multi-step chemical processes, GECIs can be modified using standard molecular biology techniques to add or modify many of their properties. (4) The GECI sequence can be fused to other proteins with breakable or permanent linkers. In this work, we used a self-cleaving linker to ensure near-stoichiometric expression of GCaMP6s to an expression marker. Many other linker types are available, which could prove useful in certain assay types. For example, we could potentially increase the sensitivity of the probe by linking the GCaMP6s directly to a target protein, thereby concentrating the probe in close proximity to the site of action. (5) Targeting sequences can be added to direct the probe to specific subcellular compartments. In this work, we had no targeting sequence so the probe was directed to the cytoplasm. In contrast, synthetic chemical indicators label subcellular compartments to varying degrees. This means that the fluorescence output is generally a mixture of responses from these different compartments. (6) GECIs are highly modular in nature, with well-defined and separable motifs for calcium binding, fluorescence properties, cellular targeting, and attachment to other proteins. This enables a near-infinite capacity for directed tuning of the properties to suit various needs. A large set of spectral properties and calcium affinities have already been discovered and characterized^8,26,45^. One example of the power of these genetic tools was the development of the calcium-measuring organelle-entrapped protein indicators (CEPIA)^23^. GCaMP2 was systematically modified to (a) add signals for ER targeting and retention, (b) alter the fluorophore to generate variations in emission/excitation spectra, and (c) reduce calcium affinity to match the higher range of calcium concentrations inside the ER. One of these indicators (R-CEPIAer) was subsequently employed in a high-throughput screening assay^24^. (7) This modularity also allows for new discoveries to be readily incorporated into existing constructs. For example, the fluorophore for GCaMP6s could easily be replaced with a red- or blue-shifted fluorophore with a reasonable expectation that the rest of the functionality of the probe would remain intact. (8) Massive libraries of mutations of an existing probe are easily made using directed or random mutagenesis to adjust functional or expression properties.

The GCaMP6s platform as described here has the potential for introduction of complications that may need to be addressed, including the following. (1) All GECIs are calcium chelators, and for fluorescence applications, need to be expressed at high levels. In some cases, constitutive expression of GECIs has been observed to have deleterious effects on cell metabolism and viability in neurons and cardiomyocytes *in vivo*^26,46^; this appears to be correlated with high expression levels and the loss of nuclear exclusion of the indicator (reviewed by Rose *et al*.^26^). Our GCaMP6s lines have been in continuous culture for 6 months with no apparent deterioration in cell health, rate of cell division, or fluorescence of the indicator. While we did not observe any issues with cell viability in these experiments, certain cell types may be more vulnerable. Such issues could be mitigated using an inducible promoter on the GECI, allowing on-demand expression of the indicator. (2) The calcium sensor of GCaMP6s is based upon calmodulin, which interacts with a wide variety of cellular targets. Overexpression of the modified CaM could potentially alter function or pharmacology of certain targets^47^. Fortunately, the importance of intracellular calcium signaling has driven the evolution of a large variety of calcium-binding proteins^48^. To date, all GECIs described in the literature are based on variants of either CaM or troponin C; development of GECIs using alternate calcium-binding motifs may provide additional advantages. (3) CaM comprises four calcium binding sites that each contribute to folding the protein into its activated state. This results in a steeper dependence of the fluorescence output over a narrower range of calcium concentrations compared to synthetic calcium indicators, which generally comprise a single calcium binding site. The Hill slope of calcium binding to CaM itself is approximately 3; mutagenesis has produced CaM-based GECIs with slopes ranging from 0.7–3.8^7^. Pharmacological concentration-response curves tend to be steeper for GCaMP6s compared to fluo-4. The relatively narrow range of useful calcium concentrations requires careful tuning of the assay conditions to avoid saturation of the indicator, and subsequent loss of pharmacological sensitivity. Future improvements to the platform could include linearizing the calcium-fluorescence curves by careful adjustment of the binding site affinities^6^ or by reducing the number of calcium binding sites^19,49^. (4) The maximal fluorescence output of 293-F cells stably expressing GCaMP6s was approximately 7X lower than 293-F cells stained with fluo-4. While the reduced signal intensity did not affect the performance of the assays described here, stable expression of GCaMP6s may provide insufficient signal in certain applications that require maximal light output. However, part of the increased fluorescence for cells labelled with fluo-4 was due to labelling of the nucleus and other intracellular organelles, which was absent in the GCaMP6s-expressing cells.

In summary, these results demonstrate that GECIs can be used as a replacement for synthetic calcium indicators in assay formats compatible with high-throughput screening. The dynamic range, assay reproducibility, and accuracy of potency measurements are comparable between GCaMP6s and fluo-4. GECIs have significant advantages over chemical indicators, including reduced cost of reagents and simplification of the assay procedure. By linking the expression of the GECI to the blasticidin selection marker using the cleavable P2A linker, we have ensured a high level of stable expression in 293-F clonal cell lines, enabling the use of these cell lines as platforms for any desired intracellular calcium assay.

## Methods

### GCaMP6s expression construct

The construct designated pGP-CMV-GCaMP6s^28^ was obtained from Douglas Kim (Addgene plasmid # 40753). The GCaMP6s open reading frame (ORF) in pGP-CMV-GCaMP6s contained an N-terminal sequence: MGSHHHHHHGMASMTGGQQMGRDLYDDDDKDLAT. This peptide contained a 6His tag, a T7 bacteriophage gene10 tag, and an XPRESS tag (tags underlined). We used polymerase chain reaction (PCR) with KOD DNA Polymerase (EMD Millipore, Darmstadt, Germany) to isolate the GCaMP6s ORF, remove the N-terminal tags, and append a BamHI site on to the C-terminus that codes for a gly-ser linker. A Kozak was added to the GCaMP6s ORF N-terminus, and the PCR product inserted into a proprietary vector driven by the CMV IE-1 promoter. The GCaMP6s ORF was fused in-frame with the BamHI gly-ser linker to the Porcine Teschovirus-1 2A (P2A) CHYSEL peptide^30^ (GATNFSLLKQAGDVEENPGP) which was further fused in frame to the *Bacillus cereus* blasticidin-resistance gene^29^ (Bsr, InvivoGen, San Diego, CA). The complete GCaMP6s-P2A-Bsr ORF was sequence-confirmed in full across the ORF (data not shown). The construct was expanded in TOP10 *E. coli* (Thermo Fisher, Carlsbad, CA) and purified with the Qiagen Mega Prep Kit (Qiagen, Valencia, CA).

### GluA1o-CACNG4 fusion protein expression construct

cDNAs for human GluA1o (Uniprot accession number P42261-1) and human TARP-γ4 (Uniprot accession number Q9UBN1) were PCR-amplified from a human brain cDNA library. To ensure a 1:1 stoichiometry of GluA1o and TARP-γ4 in the expressed channel, we fused the cDNA encoding the C-terminus of GluA1o to the cDNA encoding the N-terminus of TARP-γ4 by inserting a linker sequence encoding QQQQQQQQQQEFAT between the two full-length cDNAs^50,51^. The channels expressed with this construct appear to have similar properties to channels formed by co-expression of GluA1o with an excess of TARP^50^. Human GluA1o-γ4 fusion protein expression constructs were generated by overlapping PCR followed by cloning into pCIneo between EcoR1 and Not1 sites. The resulting plasmid was sequence-confirmed.

### TRPV1 expression construct

Guinea pig TRPV1 cDNA (Uniprot accession number Q6R5A3) was generated by reverse transcription-polymerase chain reaction from guinea pig brain mRNA^52^.

### Cell culture

293-F cells (a subclone of HEK-293 adapted for suspension culture) were obtained from ThermoFisher (Waltham, MA). 293-F cells were grown in suspension in FreeStyle™ 293 Expression Medium (Gibco, Grand Island, NY) in shake flasks at 37 °C, 8% CO2, and 120 rpm.

### Generation of stable cell lines

A clonal cell line designated 293F.GluA1o-γ4 was generated by electroporation (Gene Pulser, BioRad, Hercules, CA) of the GluA1o-γ4 expression construct into 293-F cells and selection using geneticin, followed by FACS isolation (MoFlo Astrios Cell Sorter, Beckman Coulter Life Science) of single cells. This cell line was subsequently transfected by electroporation using GCaMP6s-P2A-Bsr, followed by selection using 10 µg/mL blasticidin (Invivogen). A clonal cell line designated 293F.GluA1o-g4.GCaMP6sBlast was isolated by FACS.

A third clonal cell line designated 293F.GCaMP6s.blast was generated by electroporation (Gene Pulser, BioRad, Hercules, CA) of the GCaMP6s-P2A-Bsr expression construct into 293-F cells and selection using 10 µg/mL blasticidin, followed by FACS isolation (MoFlo Astrios Cell Sorter, Beckman Coulter Life Science) of single cells.

### Transient expression

For assays with transiently-transfected cells, cells were generated by bulk transfection. At the time of transfection, 293-F or 293F.GCaMP6s.blast cells were diluted to 1 million/mL with FreeStyle-293 medium. Transfection was performed by combining equal amounts of pAdvantage vector (Promega Corp., Madison WI) and target DNA. Total DNA was 50 µg per 40 mL transfection. The transfection reagent was Freestyle MAX (Invitrogen). Cell viability at 24 hours was above 80% for transfections to be considered successful. Cells were spun down and resuspended in HyClone™ DMEM (GE Healthcare, Little Chalfont, UK) supplemented with 10% fetal bovine serum (FBS; Omega Scientific, Tarzana, CA), then seeded into 384-well poly-D-lysine-coated plates at 10–15k cells/well at 16–24 hours after transfection, and used for assays 24–48 hours after transfection.

### Intracellular calcium assays

Cells were plated at 10–15 K cells/well in DMEM supplemented with 10% FBS into 384-well poly-D-lysine-coated plates 24–48 hours prior to the assay. Cell plates were washed with wash buffer (135 mM NaCl, 4 mM KCl, 1 mM CaCl_2_, 1 mM MgCl_2_, 5 mM glucose, 10 mM HEPES, pH 7.4, 300 mOs) using an EL405 plate washer (Biotek, Winooski, VT).

For assays based on the synthetic indicator, the cells were loaded with fluo-4 Direct (Invitrogen) at 20X dilution from stock, with 1.25 mM probenecid (Setareh Biotech, Eugene, OR). In this kit, fluo-4 is applied as the acetoxymethyl ester. Cells were incubated with dye at 37 °C for 30 minutes, followed by 30 minutes at room temperature. Cells were then washed with assay buffer immediately prior to the FLIPR assay. The composition for the assay buffer was the same as the wash buffer, with variations in the calcium concentration (specified in the Results section).

Fluorescence was monitored during the addition of reagents using a FLIPR Tetra^®^ (Molecular Devices, Sunnyvale, CA) with a 470–495 nm excitation source and a 515–575 nm bandpass emission filter. Assays were performed in single-addition mode. Test compounds known to be antagonists or positive allosteric modulators (PAMs) were either added to the wells 60 minutes prior to the assay, or added at the same time as the agonist (see details for each experiment). During the assay, fluorescence was monitored for ten seconds prior to the addition of agonist to ensure stable baselines. For time course measurements, the fluorescence was normalized to the baseline fluorescence *F*_0_ (fluorescence measurement at the first time point).

The negative control wells had no added compounds, and the positive control wells were treated with a blocking concentration of a full antagonist. All wells contained 0.1% DMSO. For the AMPA receptor assays, the positive control was 50 µM CP-465022^53^. For the muscarinic receptor assays, the positive control was 3 µM scopolamine. For the TRPV1 assays, the positive control was 10 µM capsazepine^54^.

The response *R* in each well was the fluorescence *F* in the measurement window, normalized to the mean fluorescence of negative ($\bar{N}\pm {\sigma }_{N}$) and positive ($\bar{P}\pm {\sigma }_{P}$) control wells (mean ± standard deviation (SD)) in the same measurement window:

1 $${R}=\frac{{F}-\bar{{N}}}{\bar{{P}}-\bar{{N}}}$$

Assay quality was assessed for each assay plate by calculating the *z*′ factor^55^. Under ideal conditions, the assay quality parameter *z*′ = 1, while *z*′ < 0 indicates excessive scatter in the controls:

2 $${z}{^{\prime} }=1-\frac{3({{\sigma }}_{{P}}+{{\sigma }}_{{N}})}{|\bar{{P}}-\bar{{N}}|}.$$

For potency determinations, test compounds were prepared at a range of concentrations by serial dilution. The normalized responses (*R*) as functions of the test compound concentrations (*x*) were fitted to a four-parameter Hill function:

3 $${R}={{A}}_{2}+({{A}}_{1}-{{A}}_{2})/(1+{({x}/{{x}}_{0})}^{{p}})$$

The fitted parameter corresponding to the midpoint (*x*_0_) was taken to be the potency of the compound: for antagonists (IC_50_; 50% inhibitory concentration); for PAMs and agonists (EC_50_; concentration achieving 50% maximal effect). Potency is expressed as ${{\rm{pIC}}}_{50}=-\,{\mathrm{log}}_{10}({{\rm{IC}}}_{50}[{\rm{M}}])$, or ${{\rm{pEC}}}_{50}=-\,{\mathrm{log}}_{10}({{\rm{EC}}}_{50}[{\rm{M}}])$.

The dynamic range for a fluorescent indicator is typically defined as $DR=\,{F}_{max}/{F}_{min}$, where *F*_*max*_ and *F*_*min*_ are the signals measured in calcium-saturated and calcium-depleted conditions, respectively. In an HTS setting, zero-calcium conditions are rarely used, and resting levels of intracellular calcium can be within the sensitivity range of the indicator. Therefore, a more-relevant measure is the effective dynamic range:

4 $${D}{{R}}_{{eff}}=({{F}}_{\infty }-{{F}}_{1})/{{F}}_{1}={{F}}_{\infty }/{{F}}_{1}-1$$

Here, *F*_∞_ is the maximal fluorescence signal when the cells of interest are challenged with a treatment expected to achieve near-saturating calcium conditions, and *F*_1_ is the fluorescence when the cells are unchallenged or treated with a fully-inhibited control. Preferably, *F*_∞_ should be measured using a receptor-mediated pathway. This enables measurement only of those cells that comprise both the target protein and the indicator. To account for potential dye bleaching or addition artifacts, *F*_1_ is measured within the same time window as the normal assay response. In many assays, *F*_1_ may also be used as either the negative or positive control response. Both measures incorporated in Equation 4 are sensitive to instrumental features (imperfect optics, background fluorescence of the multiwell plates, etc.) and assay-specific features (background fluorescence of the cells or other reagents, cell-to-cell variation of the indicator concentration, etc.).

The assay dynamic range is calculated as:

5 $${D}{{R}}_{{assay}}=({{F}}_{2}-{{F}}_{1})/{{F}}_{1}={{F}}_{2}/{{F}}_{1}-1$$

Here, *F*_2_ is the fluorescence for an uninhibited control. In many assays, *F*_2_ may also be used as a positive or negative control.

### Imaging

Cells were plated onto 384-well poly-D-lysine-coated plates at 10–15 K cells/well 24 hours prior to imaging. Nuclei were labelled with Hoechst 33342 (Thermo Fisher, Waltham, MA) at a final dilution of 16.7 μM. Cells were treated as described in the Results section 5 minutes prior to imaging. Confocal imaging of live cells was performed using a Zeiss LSM 700 microscope. GFP and fluo-4 were excited by a 488 nm laser (emission filter 492LP) and Hoechst 33342 by a 405 nm laser (emission filter 590SP) with a dwell time of 3.15 μsec and a line averaging setting of 2. Laser power and detector gain were set to levels just below saturation of the detector for each dye based upon the images of cells treated with ionomycin. Tracks were acquired sequentially to avoid bleed-through. 1024 × 1024 pixel (159.89 × 159.89 mm) images or 512 × 512 pixel (159.73 × 159.73 × 16.25 mm) z-stacks were captured at a depth of 12 bits. Z-stacks were processed as orthogonal projections to allow determination of calcium indicator/nuclear label colocalization. All images were exported as 2-channel 8-bit RGB TIF files, then cropped for final presentation in Adobe Photoshop.

### Flow Cytometry

Assay buffer was wash buffer adjusted to 4 mM CaCl_2_. Cells were suspended at a density of 1–3 million cells/mL in assay buffer. For those cells labelled with fluo-4, Fluo-4 Direct (Invitrogen) was added to the suspension at 20X dilution from stock, with 1.25 mM probenecid (Setareh Biotech). Cells were incubated 60 minutes at room temperature on a rocker, and then washed with assay buffer. Pharmacological treatments were added 2–3 minutes prior to cytometry in a Moxi GO II (ORFLO Technologies, Ketchum, ID). Fluorescence was detected using 488 nm laser excitation, 525/45 nm emission filter, and Very Low Gain for the detector. Particle size was detected by the Coulter method; particles smaller than 9 µm diameter were considered debris and gated out. Data analysis was performed using FlowJo V10 (Ashland, OR), with fluorescence intensities reported in Arbitrary Fluorescence Units (AFU). Non-linear least squares fitting of histograms of the log-transformed fluorescence intensities was performed using Origin Pro 2017.

### Test Compounds

Ionomycin, carbachol, oxotremorine, pirenzepine, methoctramine, 4-Diphenylacetoxy-N-methylpiperidine methiodide (4-DAMP), L-glutamate, capsaicin, dihydrocapsaicin, and ruthenium red were purchased from Sigma-Aldrich (St. Louis, MO).

Atropine, acetylcholine, 1-(4-Aminophenyl)-3-methylcarbamyl-4-methyl-3,4-dihydro-7,8-methylenedioxy-5H-2,3-benzodiazepine hydrochloride (GYKI-53655), 3-(2-Chlorophenyl)-2-[2-[6-[(diethylamino)methyl]-2-pyridinyl]ethenyl]-6-fluoro-4(3H)-quinazolinone hydrochloride (CP-465022), talampanel, 2,3-Dioxo-6-nitro-1,2,3,4-tetrahydrobenzo[f]quinoxaline-7-sulfonamide (NBQX), kainic acid, (S)-α-Amino-3-hydroxy-5-methyl-4-isoxazolepropionic acid ((S)-AMPA), (R)-AMPA, domoic acid, arvanil, N-oleoyldopamine (OLDA), capsazepine, 4-(3-Chloro-2-pyridinyl)-N-[4-(1,1-dimethylethyl)phenyl]-1-piperazinecarboxamide (BCTC), 4′-Chloro-3-methoxycinnamanilide (SB-366791), and (2E)-N-(2,3-Dihydro-1,4-benzodioxin-6-yl)-3-[4-(1,1-dimethylethyl)phenyl]-2-propenamide (AMG9810) were purchased from Tocris (Minneapolis, MN).

Perampanel was purchased from AlsaChim (Illkirch-Graffenstaden, France).

Cyclothiazide was purchased from Abcam (Cambridge, MA). N-(4-[1-Methyl-2-(propane-2-sulfonylamino)-ethyl]-phenyl)-benzamide (LY-395153) was purchased from Diverchim (Roissy, France).

5-[2-chloro-6-(trifluoromethoxy)phenyl]-1,3-dihydrobenzimidazol-2-one (JNJ-55511118), 2-(3-chloro-2-(2-oxo-2,3-dihydro-1H-benzo[d]imidazol-5-yl)phenyl)acetonitrile (JNJ-56022486), 4-[3-(Trifluoromethyl)pyridin-2-yl]-N-[5-(trifluoromethyl)pyridin-2-yl]piperazine-1-carboxamide (JNJ-17203212), and 2-(2,6-dichloro-benzyl)-thiazolo[5,4-d]pyrimidin-7-yl]-(4-trifluoromethyl- phenyl)-amine (JNJ-39729209) were synthesized in-house^35,51,56^.
